# Chemotaxis overrides the killing response in alloreactive CTLs, providing vascular immune privilege during cellular rejection

**DOI:** 10.1172/JCI155191

**Published:** 2025-05-22

**Authors:** Thomas Barba, Martin Oberbarnscheidt, Gregory Franck, Chantal Gao, Sebastien This, Maud Rabeyrin, Candice Roufosse, Linda Moran, Alice Koenig, Virginie Mathias, Carole Saison, Valérie Dubois, Nicolas Pallet, Dany Anglicheau, Baptiste Lamarthée, Alexandre Hertig, Emmanuel Morelon, Arnaud Hot, Helena Paidassi, Thierry Defrance, Antonio Nicoletti, Jean-Paul Duong Van Huyen, Yi-Chung Xu-Dubois, Faddi G. Lakkis, Olivier Thaunat

**Affiliations:** 1 CIRI, INSERM U1111, Université Claude Bernard Lyon I, CNRS UMR 5308, Ecole Normale Supérieure de Lyon, Université de Lyon, Lyon, France.; 2 Lyon-Est Medical Faculty, Claude Bernard University (Lyon 1), Lyon, France.; 3 Department of Internal Medicine Hospices Civils de Lyon, Edouard Herriot Hospital, Lyon, France.; 4 Department of Surgery, Thomas E. Starzl Transplantation Institute, University of Pittsburgh School of Medicine, Pittsburgh, Pennsylvania, USA.; 5 INSERM U 1148, Laboratory of Vascular Translational Science, Paris, France.; 6 Department of Pathology Hospices Civils de Lyon, Bron, France.; 7 Department of Medicine, Imperial College, London, United Kingdom.; 8 North West London Pathology, Imperial College Healthcare NHS Trust, London, United Kingdom.; 9 Hospices Civils de Lyon, Edouard Herriot Hospital, Department of Transplantation, Nephrology and Clinical Immunology, Lyon, France.; 10 French National Blood Service (EFS), HLA Laboratory, Décines-Charpieu, France.; 11 Laboratory of Biochemistry, Assistance Publique-Hôpitaux de Paris, Georges Pompidou Hospital, Paris, France.; 12 Department of Nephrology and Kidney Transplantation, Necker Hospital, Assistance Publique-Hôpitaux de Paris, Paris, France.; 13 Institut Necker Enfants-Malades, INSERM U1151, Paris, France.; 14 Kidney Transplantation, AP-HP, Tenon Hospital, Paris, France.; 15 Pathology Laboratory, Hôpital Necker-Enfants Malades, AP-HP, Paris, France.

**Keywords:** Immunology, Nephrology, Transplantation, Cellular immune response, Chemokines, Endothelial cells

## Abstract

Graft endothelial cells (ECs) express donor alloantigens and encounter cytotoxic T lymphocytes (CTLs) but are generally spared during T cell–mediated rejection (TCMR), which predominantly affects epithelial structures. The mechanisms underlying this vascular immune privilege are unclear. Transcriptomics analyses and endothelial-mesenchymal transition assessments confirmed that the graft endothelium was preserved during TCMR. Coculture experiments revealed that endothelial and epithelial cells were equally susceptible to CTL-mediated lysis, ruling out cell-intrinsic protection. Intravital microscopy of murine kidney grafts and single-cell RNA-Seq of human renal allografts demonstrated that CTL interactions with ECs were transient compared with epithelial cells. This disparity was mediated by a chemotactic gradient produced by graft stromal cells, guiding CTLs away from ECs toward epithelial targets. In vitro, chemotaxis overrode T cell receptor–induced cytotoxicity, preventing endothelial damage. Finally, analysis of TCMR biopsies revealed that disruption of the chemotactic gradient correlated with endothelialitis lesions, linking its loss to vascular damage. These findings challenge the traditional view of cell-intrinsic immune privilege, proposing a cell-extrinsic mechanism, in which chemotaxis preserves graft vasculature during TCMR. This mechanism may have implications beyond transplantation, highlighting its role in maintaining vascular integrity across pathological conditions.

## Abstract 2

This study dissects the mechanisms of endothelial preservation during cellular rejection, and identifies chemotaxis as the driver of a new form of immune privilege.

## Graphical abstract

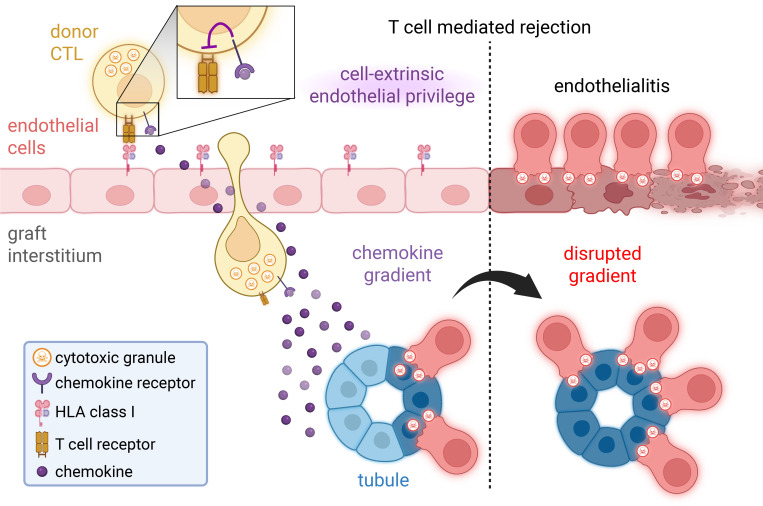

## Introduction

In solid organ transplantation, the genetic differences between the donor and recipient, especially at the level of the highly polymorphic molecules from the major histocompatibility complex (i.e., HLA) in patients, are unavoidably recognized by the adaptive immune system of the recipient. In response, the adaptive immune system develops effector mechanisms responsible for the destruction of the transplanted organ, a process known as “rejection.” Depending on the nature of the dominant immune effector mechanism, one currently distinguishes 2 main types (sometimes associated) of rejections: cellular or T cell–mediated rejection (TCMR) and antibody-mediated rejection (AMR) (1).

Donor-specific antibodies are largely sequestrated in the recipient’s circulation due to their size (2). For this reason, grafted allogeneic tissue such as islets of Langerhans, which are neovascularized by recipient cells (2), are protected from the deleterious effect of alloantibodies (2, 3). In contrast, in the case of solid organ transplantation, the vasculature of the organ is anastomosed to that of the recipient. The graft vasculature is therefore the only accessible target for circulating donor-specific antibodies, the binding of which triggers lesions through activation of the classical complement pathway (4) and/or the recruitment of the host’s innate immune effectors responsible for antibody-dependent cell cytotoxicity (5–7). In contrast, the interstitium and tubular epithelial cells of the graft remain essentially preserved during typical antibody-mediated rejection (1).

During TCMR, recipient CD8^+^ cytotoxic T lymphocytes (CTLs), previously primed in the secondary lymphoid organs by antigen-presenting cells (8), infiltrate graft interstitium after vascular transmigration and damage the tubular epithelial structures (1). Strikingly, while experimental studies have demonstrated the primary role of cognate antigen presented by donor endothelial cells (ECs) in the migration of alloreactive T cells across the vascular barriers (9), the graft vasculature is usually spared during TCMR, except in the most severe cases (1).

This concept of damage compartmentalization that distinguishes the 2 types of allograft rejection (vascular lesions in AMR vs. epithelial lesions in TCMR), although routinely exploited by pathologists and clinicians caring for patients who have undergone transplantation, is not yet clearly understood. In this translational study, mixing various analytical methods, including transcriptomics and intravital microscopy, as well as newly developed in silico and in vitro models of murine and human samples, we investigated the mechanisms by which the endothelium of the graft microvasculature evades the cytotoxicity of alloreactive CD8^+^ T cells.

## Results

### Opposite profiles of damage compartmentalization in AMR and TCMR.

**Figure 1:**
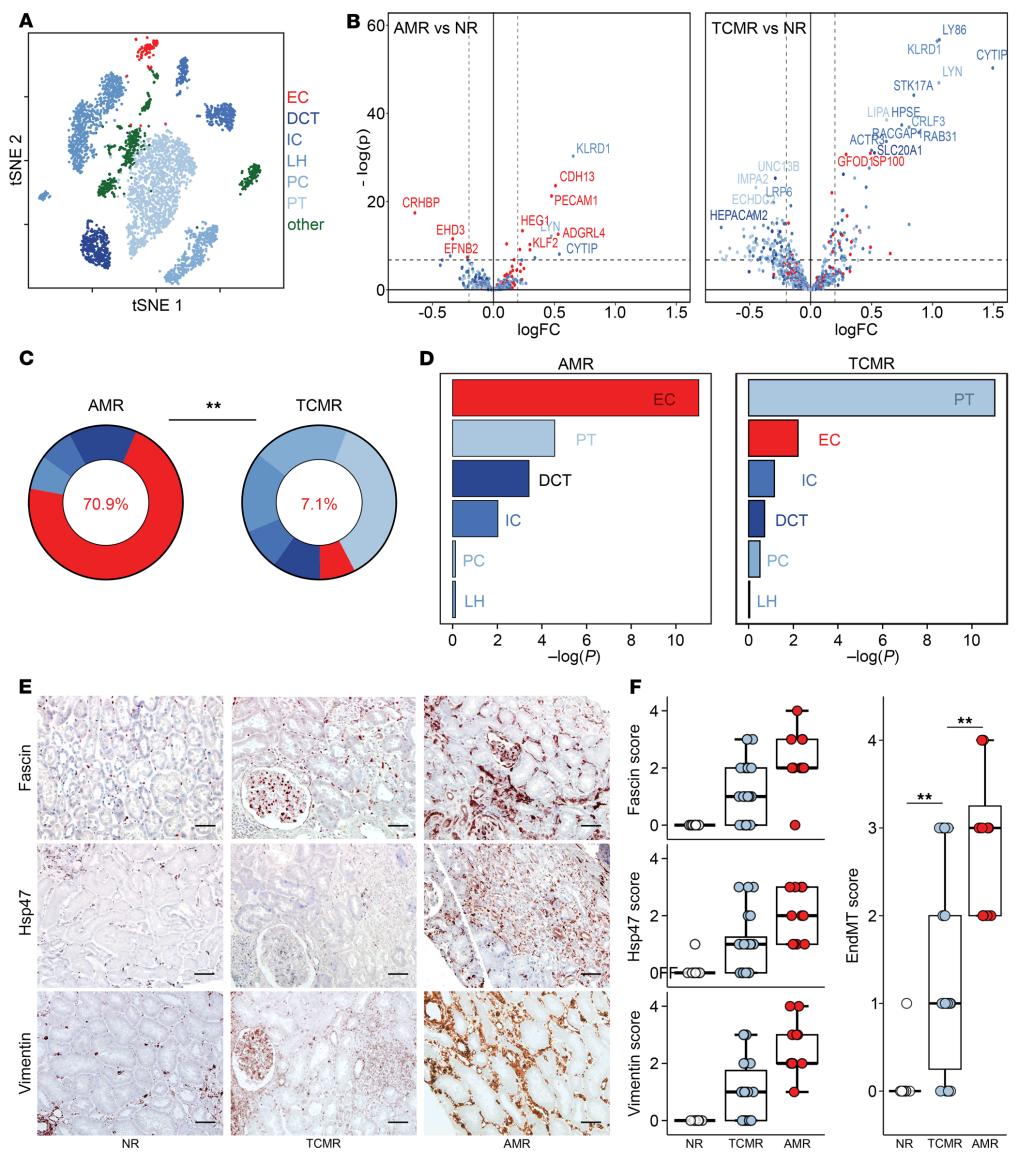
Opposite profiles of damage compartmentalization in AMR and TCMR. (**A**) Unsupervised clustering analysis of healthy donor kidney scRNA-Seq data was performed to determine endothelial (red) and tubular epithelial (blue) lineage-specific gene lists. (**B**–**D**) Comparison of transcriptomics profiles of AMR and TCMR samples. Microarray analyses of 105 AMR, 67 TCMR, and 501 NR graft biopsies were used to establish the transcriptomics profiles of AMR and TCMR using NR samples as a reference. DEGs in AMR and TCMR were filtered out using the endothelial and tubular epithelial lineage gene lists established in **A**. (**B**) Volcano plots showing the change in expression of endothelial and epithelial lineage genes in AMR and TCMR samples. (**C**) Comparison of the proportions of epithelial and endothelial genes in DELGs. **P < 0.01, by χ^2^ test. (**D**) GSEA of endothelial (red) and tubular epithelial (blue tones) lineage gene sets in AMR and TCMR samples using NR as a reference. (**E** and **F**) Expression of EndMT markers (fascin, hsp47, vimentin) in biopsies of transplanted kidneys with no rejection features (NR, negative control, *n* = 9), TCMR (*n* = 22), or AMR (*n* = 12). (**E**) Representative microscopy features (scale bars: 40 μm). (**F**) Each EndMT marker and the EndMT score were assessed by a trained nephropathologist blinded to the clinical data using a semiquantitative scale in the PTC ECs identified using a CD34-based mask. Data represent the mean ± SEM. **P < 0.01, by unpaired, 2-sample Wilcoxon test.

The rejection process dramatically affects the transcriptome of targeted graft cells by either causing the loss of gene expression essential for normal cellular function (cell dedifferentiation) or by promoting the upregulation of stress-related genes that protect against damage (10). This concept was leveraged to compare the intensity of damage in endothelial and tubular epithelial compartments of the graft in the 2 main endotypes of rejections. First, the single-cell RNA-Seq (scRNA-Seq) dataset of a healthy donor kidney (11) was used to establish a series of gene sets specifically expressed by each kidney cell type (hereafter referred as lineage genes; Figure 1A). Then, 2 microarray datasets (GSE36059 and GSE48581) (12, 13) of kidney transplant biopsies with pure AMR (*n* = 105) or TCMR (*n* = 67) or no rejection (NR, *n* = 501) were analyzed, and the respective proportions of endothelial and tubular epithelial lineage genes that were differentially expressed (differentially expressed lineage genes [DELGs]) were determined. The volcano plots shown in Figure 1B synthesize the variation of lineage gene expression in AMR and TCMR samples in comparison with nonrejected graft (NR) samples, respectively. While endothelial genes represented the vast majority (70.9%) of the DELGs in AMR biopsies, they were only minimally represented (7.1%, *P* < 0.001) in the DELGs of TCMR biopsies (Figure 1, B and C), thus suggesting that, while graft endothelium is the main target of AMR, it remains largely preserved during TCMR. In complete opposition to endothelial genes, the expression of tubular epithelial genes was much more affected by TCMR than AMR (92.9% vs. 29.1% of DELGs, respectively; *P* < 0.001; Figure 1, B and C).

Gene set enrichment analyses (GSEA) confirmed these results showing that endothelial and proximal tubule (PT) epithelial genes were the most enriched in AMR- and TCMR-associated DELGs, respectively (Figure 1D).

An additional analysis was performed using scRNA-Seq data of 8 kidney graft biopsies (E-MTAB-12051): 3 AMR, 4 NR, and 1 TCMR. Single cells (*n* = 27,412) were pooled, and uniform manifold approximation and projection (UMAP) was applied to reduce dimensionality, revealing 8 clusters (Supplemental Figure 1A; supplemental material available online with this article; https://doi.org/10.1172/JCI155191DS1), with cluster 3 representing ECs (identified by PECAM-1 [Platelet endothelial cell adhesion molecule-1] expression, Supplemental Figure 1B). Transcriptomics comparisons were made using volcano plots for AMR versus NR (Supplemental Figure 1C) and TCMR versus NR (Supplemental Figure 1D). GSEA analysis further explored cell damage and stress pathways. Results confirmed that ECs in AMR grafts showed greater damage, whereas TCMR grafts appeared preserved, supporting our initial findings.

### Graft endothelium is spared by alloreactive T cells during TCMR.

Endothelial-mesenchymal transition (EndMT), during which injured ECs adopt the expression of mesenchymal cell–specific proteins, is a validated marker for endothelial damage (14, 15). To confirm that graft ECs are spared by alloreactive T cells during TCMR, we performed quantitative analysis of the expression of 3 EndMT proteins (fascin, hsp47, and vimentin) by graft endothelium of kidney transplant biopsies with AMR (*n* = 12; used here as a positive control; see previous paragraph) and TCMR (*n* = 22). Nine systematic kidney graft biopsies, devoid of rejection lesions, were included in the analysis as negative controls. In line with our hypothesis, all 3 of the EndMT markers were expressed at significantly higher levels, and the EndMT score was higher for the vasculature of grafts with AMR than for those with TCMR (Figure 1, E and F). Although a moderate level of vascular damage was observed in TCMR compared with negative controls, these results are in line with the molecular data and confirm that graft ECs were only marginally affected during TCMR.

### The recipient’s T cells establish interactions with both endothelial and tubular epithelial cells of the graft.

**Figure 2:**
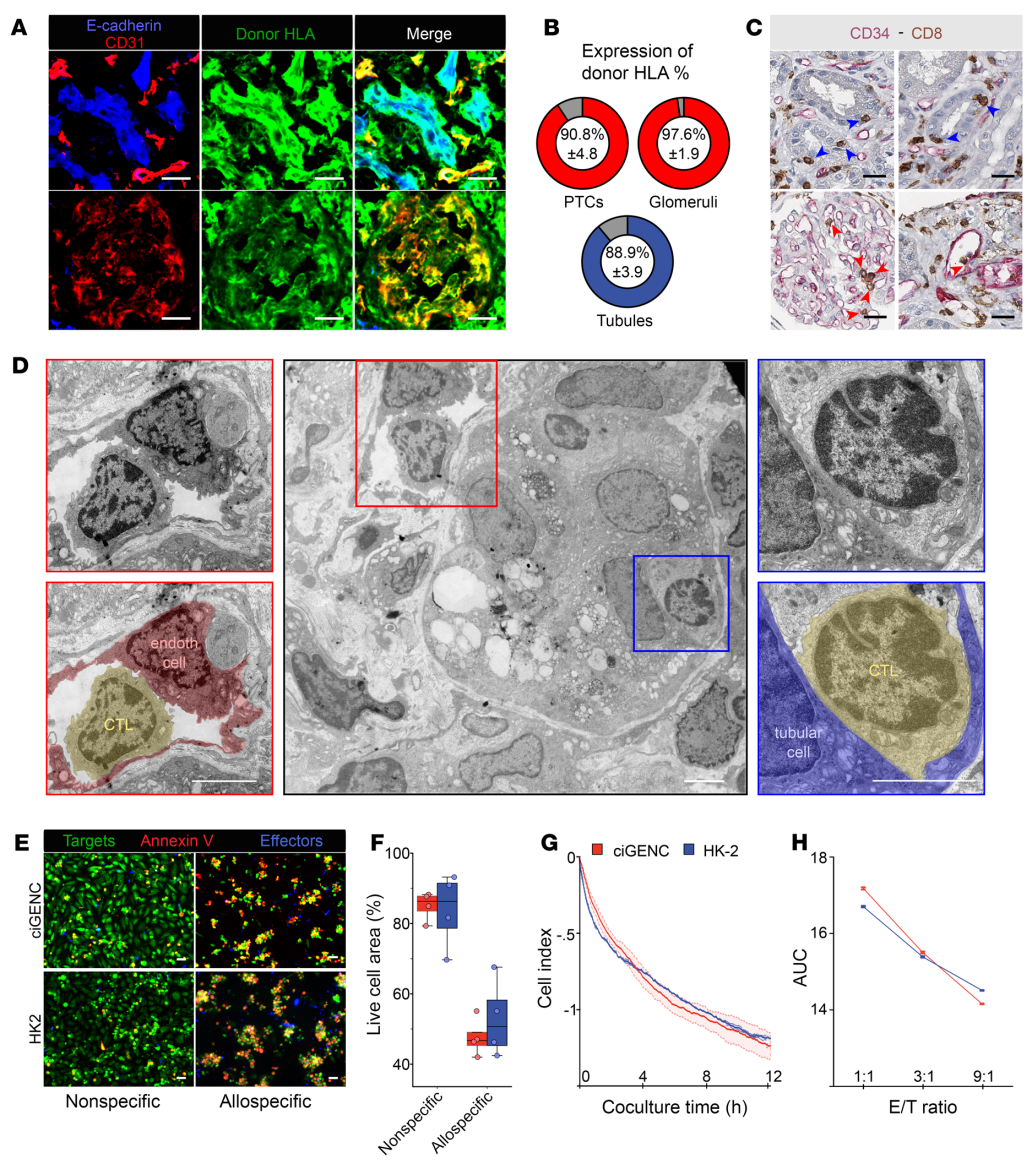
Alloreactive CTLs interact with allogeneic ECs in vivo and mediate their destruction in vitro. (**A** and **B**) Confocal microscopy analyses of renal allograft biopsies with TCMR. (**A**) The donor origin of graft cells was confirmed by the expression of donor-specific mismatched HLA A24 molecules (green) on CD31^+^ (red) ECs (glomerular and peritubular capillaries) as well as on E-cadherin^+^ (blue) tubular epithelial cells (scale bars: 20 μm). (**B**) Percentage of CD31^+^ peritubular capillary and glomerular ECs (upper row, red) and tubular epithelial cells (lower row, blue) expressing donor-specific HLA A24 molecules. Results are from the analysis of distinct fields of 4 independent biopsies (mean ± SEM). (**C**) IHC analyses of renal allograft biopsies with TCMR. CD8^+^ CTLs (brown) interacted with graft tubular epithelial cells (upper row) as well as the CD34^+^ ECs (red; lower row) of glomerular (left panels) and peritubular (right panels) capillaries (scale bars: 20 μm). (**D**) Electron micrographs of the interactions of CTLs with the various compartments of a renal allograft (middle panel). Magnification of the interactions of CTLs with, respectively, the EC (endoth cell) of a peritubular capillary (left panels) and a tubular epithelial cell (right panels) is shown (scale bars: 5 μm). (**E**–**H**) Quantification of allospecific, CTL-mediated killing of glomerular ECs (ciGENC, red) and PT epithelial cells (HK-2, blue). (**E**) Target cell destruction by nonspecific or allospecific CTLs was assessed using time-lapse microscopy. The decrease in live cell area (calcein, green) and acquisition of the apoptosis marker (annexin V, red) were quantified. Representative images from the end of cocultures are shown (scale bar: 50 μm). (**F**) Quantification of cell destruction from 2 independent time-lapse experiments (median ± IQR ). Significance was determined by unpaired, 2-sample Wilcoxon test. (**G**) Kinetics monitoring of ciGENC (red) and HK-2 (blue) target cell destruction by allospecific CTLs based on impedance reduction in culture wells. Impedance values were normalized to control conditions with nonspecific CTLs. (**H**) AUC of the impedance values for ciGENC and HK-2 cocultures at varying effector/target ratios. Results from 2 independent experiments are shown. Significance was determined by 1-way ANOVA.

Two trivial explanations could account for the preservation of ECs during TCMR. First, the ECs of the graft could have been completely replaced by cells originating from the recipient, as observed in aortic interposition, a reductionist murine experimental model in which recipients are not treated with immunosuppressive agents (16, 17). To test this theory, we determined the origin of graft ECs on the basis of donor/recipient HLA expression in kidney graft biopsies with TCMR. Infiltrating immune cells (of recipient origin; Supplemental Figure 1) and tubular epithelial cells (of donor origin; Supplemental Figure 2) were used as controls. Ruling out the chimerism hypothesis, immunofluorescence analyses revealed that, as epithelial cells, more than 90% of graft ECs (both in the peritubular capillaries and the glomeruli) expressed donor HLA molecules (Figure 2, A and B, upper and lower rows).

Another explanation for the preservation of ECs during TCMR would be the lack of interaction between allogeneic graft microvasculature and the recipient’s T cells. However, this hypothesis is not supported by the fact that, besides the expected interactions between the recipient’s T cells and tubular epithelial cells of the graft (Figure 2C, upper row), most TCMR biopsies also show features of microvascular inflammation at the level of the peritubular capillaries, including the presence of CD8^+^ T cells adhering to graft ECs (Figure 2C, lower row). This finding has already been reported by several independent groups (18) and is included in the Banff histopathological consensus for interpretation of kidney graft biopsies (1).

Transmission electron microscopy (TEM) analysis of TCMR biopsies revealed additional ultrastructural information on the interactions between alloreactive T cells and graft cells (Figure 2D, middle panel). Observation of serial sections confirmed that mononuclear round cells with cytoplasmic granules, likely cytotoxic lymphocytes, established close interactions with both ECs of peritubular capillaries and tubular epithelial cells (Figure 2D, left and right panels). However, although these cellular interactions were indistinguishable with this technique, they had a completely different effect on the ECs that remained relatively unharmed on one hand, and the tubular epithelial cells, which showed dilatation of ER and accumulation of vacuolated lysosomes (Figure 2D, middle panel).

### ECs are not intrinsically protected against alloreactive T cell cytotoxicity.

To test the theory of a cell-intrinsic molecular mechanism protecting ECs during TCMR, an in vitro model was set up (Supplemental Figure 3), in which human alloreactive CTLs were cocultured either with the human glomerular EC line ciGENC (19) or with the human tubular epithelial cell line HK-2 (20) (Supplemental Figure 3A). Both cell lines express the HLA-A2 molecule (Supplemental Figure 3A), making them susceptible targets for alloreactive CD8^+^ T cells. Alloreactive CTLs were generated by coculturing purified CD8^+^ T cells from HLA A2-negative healthy volunteers with the HLA A2–expressing SAL-A2 cell line (21) (Supplemental Figure 3B). In contrast with unspecific CD8^+^ effector T cells generated by stimulation with anti-CD3/anti-CD28 mAbs (negative controls), allospecific CD8^+^ T cells efficiently killed the ciGENC (Supplemental Figure 3, C and D) in a strictly HLA class I–dependent manner (Supplemental Figure 3E).

The same model was used to compare the susceptibility of microvascular ECs and tubular epithelial cells with the cytotoxicity mediated by alloreactive CTLs. Neither time-lapse fluorescence microscopy (Figure 2, E and F), nor real-time impedancemetry (Figure 2, G and H) analyses demonstrated any survival advantage of microvascular ECs over tubular epithelial cells, at any of the effector/target ratios (Figure 2H).

These data suggest that the mechanism by which the graft vasculature escapes the destruction mediated by alloreactive CTLs during TCMR is not an intrinsic property of ECs.

### Difference in the dynamics of interactions between alloreactive T cells and endothelial versus tubular epithelial cellular targets.

**Figure 3:**
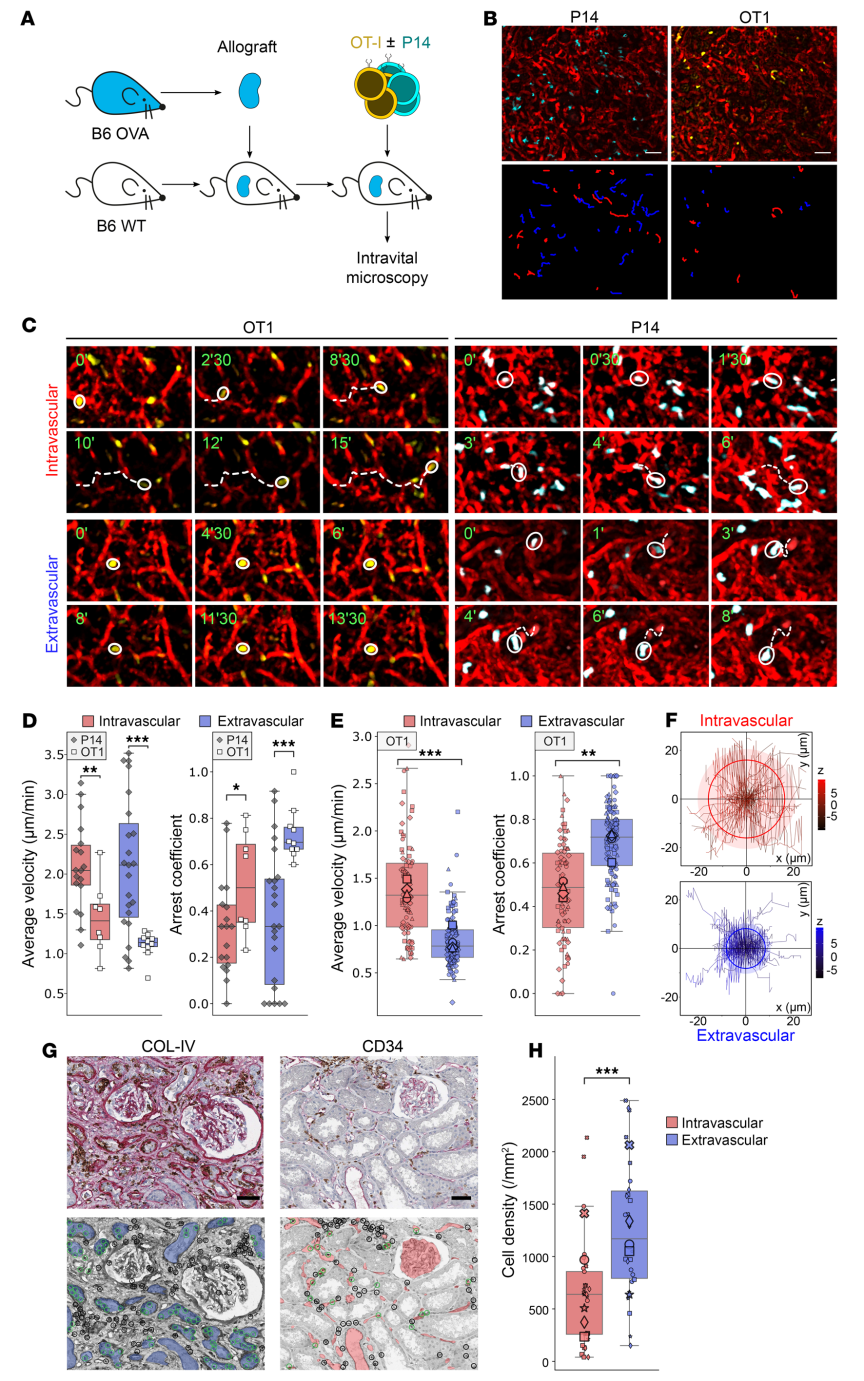
Distinct contact durations of alloreactive CTLs with endothelial and tubular epithelial cell targets. (**A**) Schematic representation of the murine model of TCMR of renal allografts. (**B** and **C**) Representative findings of intravital microscopy analysis of OVA-specific OT-I (yellow) or control P14 (cyan) CTLs trafficking within B6-OVA renal allografts. The vascular compartment is identified by fluorescent dextran (red). (**B**) Global view. The tracks of the cells were color-coded according to their position: intravascular (red) or extravascular (blue). Scale bars: 50 μm. (**C**) Representative behavior of OT-I (left columns) and P14 (right columns) cells in the intravascular (upper rows) and extravascular (lower rows) compartments (scale bars: 10 μm; time stamps format = mm:ss). (**D**–**F**) Comparison of the trafficking behavior of OT-I and P14 cells in intravascular (red) and extravascular (blue) compartments of the graft. Results in **D** and **E** represent independent experiments. (**D**) OVA-specific OT-I and control P14 CTLs were cotransferred into a mouse that had received a B6-OVA renal allograft (*n* = 1). Each symbol corresponds to a tracked cell. (**E**) OVA-specific OT-I CTLs were transferred alone into mice that had received a B6-OVA renal allograft (*n* = 2 experiments involving 2 animals each; each shape represents an individual animal). Individual cells are represented by a small symbol, and the larger symbol is the mean for the animal. Data represent the mean ± SEM. **P < 0.01 and ***P < 0.001, by 2-sided Student’s *t* test. (**F**) Overlay of individual OT-I T cell tracks plotted after aligning their starting positions. Cells were tracked over a 30-minute period in the intravascular (red) or extra vascular (blue) compartments. (**G** and **H**) Quantification of infiltrating CTLs in vascular and tubular epithelial compartments of 5 renal allograft biopsies with TCMR. (**G**) Computer-assisted quantification of CTLs (CD8^+^, brown) in the tubular epithelial (COL-IV, red, left column) and vascular (CD34^+^, red, right column) compartments of rejected renal allografts. CTLs were automatically counted within (green circles) and outside (black circles) each compartment (scale bars: 50 μm). (**H**) The density of CTLs in intravascular (red) and tubular epithelial (extravascular, blue) compartments was compared. E/T, effector/target ratio. ***P < 0.001, by Wilcoxon signed-rank test.

In order to better understand the mechanism by which a graft’s ECs resist the destruction mediated by alloreactive T cells, we compared the dynamics of the interactions between the host’s T cells and the 2 types of the graft’s cellular targets (endothelial and tubular epithelial cells) in a murine kidney transplantation model (Figure 3A). Briefly, C57BL/6 mice were used as recipients of a kidney transplant from an OVA-transgenic C57BL/6 donor mouse (9). In this model, OVA expressed on the surface of graft cells serves as a minor histocompatibility antigen (22). Kidney graft recipient mice were adoptively transferred with fluorescently labeled OT-I T cell receptor–transgenic (TCR-transgenic) CD8^+^ CTLs. These cells specifically recognize OVA residues 257–264 presented in the context of H2-Kb, enabling them to detect the graft’s minor histocompatibility antigen OVA via the indirect pathway (23). In some experiments, fluorescently labeled P14 TCR-transgenic CD8^+^ CTLs (specific to the LCMV [lymphocytic choriomeningitis virus] peptide gp33 presented in the context of H2-D^b^, used here as unspecific controls) were cotransferred with OT-I cells. The vascular compartment was identified by i.v. injection of fluorescently labeled dextran, and lymphocyte migration in the kidney transplant was monitored in real time (Supplemental Videos 1–3) by 2-photon intravital microscopy as previously described (24).

The mean distance traveled and velocity of unspecific P14 cells were significantly higher than those of OT-I–specific cells in both compartments (Figure 3, B–D). P14 cells also exhibited a lower mean arrest coefficient than did OT-I cells (Figure 3D), indicating that, while unspecific CD8^+^ T cells merely trafficked to the kidney transplant, alloreactive OT-I cells established cognate, long-lasting interactions with both endothelial and tubular epithelial cells of the graft.

A new set of experiments specifically focused on the comparison of the interactions between OT-I and the graft’s endothelial and tubular epithelial cells, respectively, revealed that the velocity and mean traveled distance of the OT-I cells were significantly greater in the intravascular compartment than in the extravascular compartment (respectively, 2.8 vs. 1.6 μm/min, *P* < 0.001, Figure 3E; and 16.0 μm vs. 8.1 μm, *P* < 0.001, Figure 3F). Furthermore, the OT-I cell arrest coefficient, a surrogate marker for the establishment of cytotoxic immunological synapses with allogeneic cellular targets (25), was significantly lower in the intravascular compartment than in the extravascular compartment (0.43 vs. 0.65, *P* < 0.001; Figure 3E).

Together, these in vivo data confirm that, while CTLs established cognate interactions with both endothelial and tubular epithelial cells of the graft, their dynamic behavior differed substantially.

### Interactions between the recipient’s T cells and the graft’s ECs in the clinic.

Intravital microscopy monitoring of interactions between alloreactive T cells and graft cellular targets is not feasible in patients. However, with the aim of validating our observations in a clinical context, we reasoned that TCMR biopsies are actually snapshots of T cells trafficking within the graft and thus might contain useful information. In order to demonstrate that the topographical distribution of CD8^+^ T cells in renal allograft reflects the duration of their interactions with the different cellular targets, we created an in silico model (Supplemental Video 4). The latter was used to analyze the effect of 3 key variables — the number of graft-infiltrating T cells, the difference in surfaces of endothelial and tubular epithelial compartments, and the duration of the interactions between alloreactive T cells and the cellular targets — on the density of T cells observed in both compartments of a biopsy. Using this in silico model, we demonstrated that when the count of T cells was normalized over the surface of the compartment, only a difference in the duration of the cellular interactions generated an unbalanced distribution of T cells independently of the time point chosen for the snapshot analysis (ANOVA, *P* = 0.0027; Supplemental Figure 4).

Using T cell density within each compartment as a surrogate for the duration of the interactions between alloreactive T cells and the cellular targets, we performed a computer-assisted analysis of TCMR biopsies. CTLs were stained with an anti-CD8 mAb, and their distribution within both endothelial and tubular epithelial compartments of the graft was analyzed. Briefly, we delineated the vascular compartment (containing endothelial targets) using CD34 staining and the tubular-epithelial compartment using anti–collagen IV (anti–COL-IV) mAb staining of the basal membrane of the tubules (Figure 3G). We determined the density of infiltrating CD8^+^ T cells in each compartment using a blob detection algorithm, with values normalized to the surface area (Figure 3G). The density of CTLs was significantly higher in the tubular epithelial compartment than in the vascular compartment (Figure 3H), indirectly supporting the hypothesis that the duration of the interactions of CTLs with tubular epithelial cells is longer than with ECs.

### Chemotaxis protects graft endothelium against alloreactive T cell–mediated cytotoxicity.

As demonstrated above, alloreactive T cells established cognate interactions with both endothelial and tubular epithelial cells of the graft, but with different dynamics. What is the cause of this difference, and could it explain why TCMR damage is concentrated in the epithelial compartment of the graft?

The data shown in Figure 2, E–H, demonstrate that endothelial and tubular epithelial cells were equally sensitive to alloreactive T cell–mediated cytotoxicity. These results rule out the existence of a cell-intrinsic privilege of ECs and suggest that our first model failed to capture the key parameter explaining the relative preservation of the graft vasculature during TCMR.

**Figure 4:**
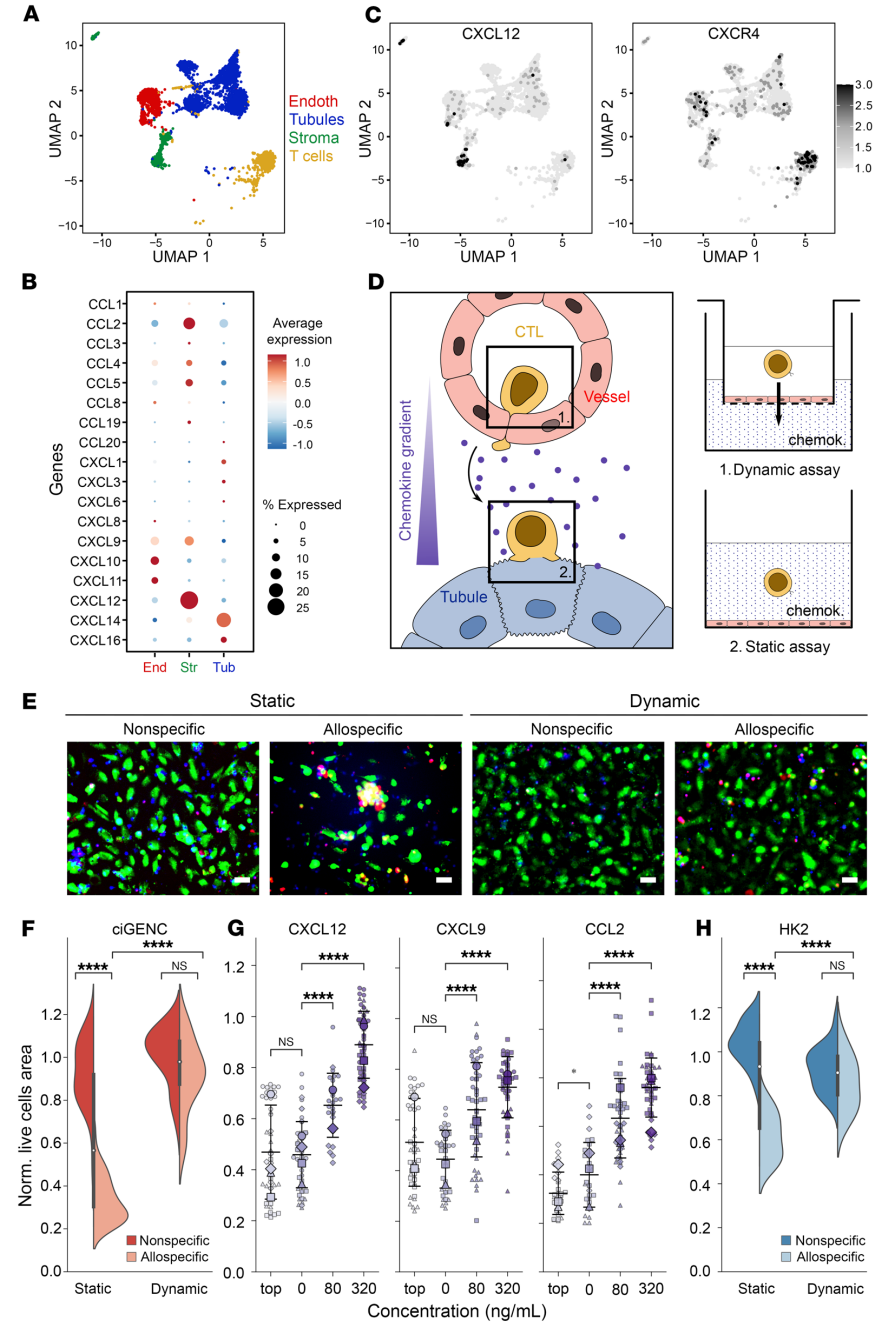
Chemotaxis protects graft endothelium against alloreactive T cell–mediated cytotoxicity in vitro. (**A**) UMAP plot of 4 cell clusters (ECs, epithelial cells, stromal cells, and infiltrating T cells) identified by scRNA-Seq analysis of a rejected kidney allograft. (**B**) Bubble plots comparing the expression of 18 cytokines genes by ECs (end), epithelial cells (tub), and stromal (str) cells of the rejected graft. Bubble size is proportional to the percentage of cells in a cluster expressing the gene, and color intensity is proportional to the average scaled gene expression level (avg. expr). (**C**) Relative expression of CXCL12 (left panel) and CXCR4 (right panel) overlaid on UMAP plot. Color intensity is proportional to average scaled gene expression. (**D**–**H**) In vitro modeling of dynamic and static CTL–target cell interactions. (**D**) Schematic representation of the dynamic and static assays. chemok., chemokine. (**E**) ciGENC target cells were cultured with 80 ng/mL CXCL12 in static (sta) or dynamic (dyn) assay, with nonspecific (left column) or allospecific (right column) CTLs (CTV stained, blue). Target cell destruction was monitored by the decrease in live cell area (calcein, green) and acquisition of the apoptosis marker (ethidium bromide, red) measured by fluorescence microscopy. Representative images at the end of cocultures are shown (scale bars: 50 μm). (**F**) Quantification of ciGENC target cell destruction. Results (mean ± SEM) of 2 independent experiments are shown. ****P < 0.0001, by unpaired, 2-sample Wilcoxon test. (**G**) The same experiments were conducted with increasing concentrations (0–320 ng/mL) of CXCL12 and other chemokines (CXCL9 and CCL2). Where indicated, the chemokine was placed in the upper (top, 320 ng/mL) chamber of the assay. Each symbol shape represents an individual experiment (CXCL12, *n* = 4; CXCL9, *n* = 3; CCL2, *n* = 3). Small symbols are replicates, and larger symbols indicate the mean. (**H**) The same experiments (*n* = 2) were performed using the tubular epithelial cell line HK2 as target cells. ****P < 0.0001, by two-sided Student’s *t* test. Data are presented as mean ± SEM (**G** and **H**).

Among the parameters that were not included in the static coculture model was chemotaxis, which contributes to the recruitment of T cells from the circulation to the rejected allograft. To identify the chemokine(s) involved in this process, we performed scRNA-Seq analysis on cells from the 3 compartments (endothelial, interstitial, and tubular) of a kidney graft undergoing T cell–mediated rejection (26). Comparison of the repertoire and intensity of chemokine production by the 3 types of graft-resident cells (Figure 4A) revealed that interstitial cells represented the main source of chemokines (Figure 4B). Among the chemokines predominantly produced by interstitial cells was CXCL9 (Figure 4B), which has been shown to be upregulated in rejected kidney allografts and at levels correlating with the intensity of interstitial inflammation and tubulitis lesions in biopsy specimens (27). In addition to CXCL9, interstitial cells alone produced CCL2 and CCL5 (Figure 4B), two chemokines critical for the recruitment of CTLs to inflammatory sites (28). However, the most striking difference between the interstitial and the 2 other graft compartments was the massive upregulation of CXCL12 expression (Figure 4, B and C [left panel in C]). T cells expressed CXCR4, the specific receptor for CXCL12, further suggesting the importance of these 2 chemokines in the recruitment of T cells within the rejected graft (Figure 4C, right panel). In line with this hypothesis, CXCL12 has previously been shown to synergize with CCL2 and CCL5 to recruit T cells to inflammatory sites (29). Furthermore, CXCL12 signaling in T cells interferes with the TCR pathway (30) and influences cytotoxic synapse duration (31). We therefore postulated that circulating alloreactive T cells may only establish short interactions with ECs of the graft because they instead favor responding to the gradient of CXCL12.

To test how chemotaxis influences alloreactive T cell–mediated cytotoxicity, we developed 2 distinct in vitro assays: a “dynamic” assay modeling conditions in the circulation and a “static” assay representing the graft’s interstitial compartment (Figure 4D). For the dynamic assay, ECs were seeded on the membrane of a Transwell insert and cultured until they reached confluence. CTLs (specific or not for the endothelial targets) were added to the top chamber, which was then placed over a culture well containing CXCL12. As expected, effector T cells responded in a dose-dependent manner to the chemokine gradient and actively migrated through the monolayer of ECs (Supplemental Figure 5). Comparison of the survival of the endothelial target cells in the static and dynamic assays confirmed that ECs were significantly less damaged by alloreactive T cells that were migrating along a CXCL12 gradient (Figure 4, E and F). Several lines of evidence indicate that this protection depends on the active migration of T cells rather than another action of CXCL12 on the biology of alloreactive T cells. Indeed, the survival of the target ECs in the upper chamber of the dynamic assay correlated with T cell migration intensity (Figure 4G, left panel), and we observed a similar dose-dependent response when using alternative CD8^+^ T cell CXC (CXCL9, Figure 4G, middle panel) or CC chemoattractants (CCL2, Figure 4G, right panel). Finally, no endothelial protection was observed when T cell migration was abrogated by adding a high dose of any of these chemokines in the top chamber of the assay (Figure 4G).

The fact that we obtained similar results when replacing ECs with tubular epithelial cells as target cells in the dynamic assay (Figure 4H) confirms the cell-extrinsic nature of this new form of immune privilege.

### Chemotaxis-dependent protection of graft endothelium persists under flow conditions.

These findings emphasize the critical role that dynamic cell interactions play in determining the fate of graft ECs upon contact with allospecific T cells migrating into the graft. A key factor that was previously unaccounted for is the influence of shear forces from blood flow, which could notably affect these interactions (32).

To investigate this, we cultured a monolayer of microvascular ECs within microfluidic channels, coated with extracellular matrix either containing or lacking CXCL12. Confluent ECs were subjected to tangential shear stress for 48 hours, leading to cell alignment in the direction of flow (Supplemental Figure 6A). Subsequently, we treated the ECs with IL-1β to upregulate E-selectin, ICAM-1, and VCAM-1, simulating the inflammatory conditions associated with graft rejection and promoting interactions with T cells (Supplemental Figure 6B). Importantly, the effect of IL-1β on endothelial activation was, if anything, increased rather than altered by the presence of CXCL12 within the extracellular matrix (Supplemental Figure 6B).

**Figure 5:**
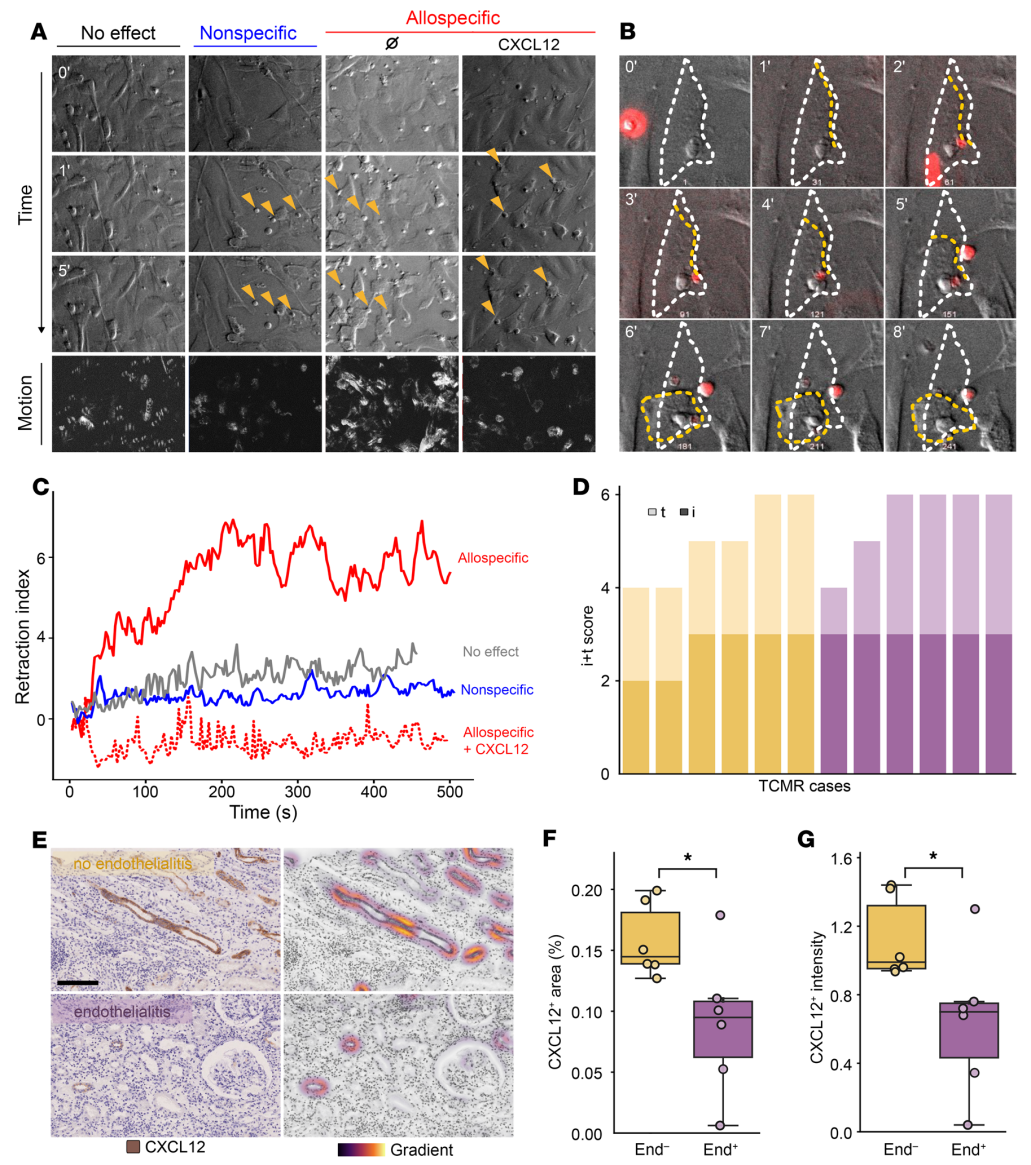
Disruption of the chemokine gradient associates with endothelial damage in clinical TCMR. (**A**–**C**) ciGENC cells were cultured to confluence under flow conditions, and their morphology was monitored in real time by video microscopy. (**A**) Representative bright-field images. The first column (No effect) shows ciGENC morphology in the absence of CTLs, while the other columns show their response to the addition of either nonspecific CTLs or allospecific CTLs. Where indicated, CXCL12 was incorporated into the coating beneath the ciGENC. Interactions between CTLs and ciGENC are marked with orange arrowheads. (**B**) Magnified time-lapse sequence illustrating the morphological changes of a ciGENC engaged by an allospecific CTL (red) in the absence of CXCL12. (**C**) Quantification of ciGENC morphological changes over time across the different experimental conditions. (**D**–**F**) Comparison of the CXCL12 gradient in 12 TCMR kidney graft biopsies with (v >0, purple, *n* = 6) or without (v = 0, orange, *n* = 6) endothelialitis (**D**) Banff i (faded color) and t (dark color) scores for each biopsy are plotted. (**E**) Representative images of CXCL12 staining (left column) (scale bar: 75 μm) and corresponding computer-assisted quantification of the chemotactic gradient (right column). Percentages of biopsy surface area (**F**) and staining intensity (**G**) for CXCL12 were compared between TCMR kidney graft biopsies with (purple, End^+^) and without (orange, End^–^) endothelialitis. *P < 0.05, by nonparametric Mann-Whitney U test. Data are presented as median ± IQR (**F** and **G**).

Next, T cells (allospecific or not) were introduced into the microfluidic circuit, and their interactions with activated endothelial targets were continuously monitored via real-time video microscopy (Figure 5A). In the absence of a CXCL12 gradient, allospecific T cells caused substantial damage to ECs, as evidenced by pronounced retraction of endothelial targets (Figure 5, A–C, see solid red line in C). In contrast, these cytotoxic effects were absent when ECs were exposed to nonspecific T cells (Figure 5, A and C, see solid blue line in C). Notably, when a CXCL12 gradient was present, the damage caused by allospecific T cells was completely prevented (Figure 5, A and C, see dashed red line in C).

These results demonstrate that, even under flow conditions, hemodynamics had a relatively minor effect on graft endothelial protection during TCMR. Instead, the presence of a chemotactic gradient, such as with CXCL12, played a pivotal role in safeguarding ECs from allospecific T cell–mediated injury.

### The disruption of the chemokine gradient is associated with endothelial damage in TCMR.

Graft ECs remain relatively preserved during TCMR compared with tubular epithelial cells, but this protection is partial. Some level of cellular damage can indeed be detected when sensitive techniques are used to compare EC status in NR versus TCMR biopsies (Figure 1). In fact, the international Banff classification of renal allograft pathology has established that some forms of TCMR are characterized by the existence of vascular damage (v lesions) (1).

On the basis of our data suggesting that graft ECs were protected from allospecific T cells migrating along a chemokine gradient, we hypothesized that the development of endothelial damage during severe TCMR may result from the disruption of this protective gradient.

To test this hypothesis, we compared the CXCL12 gradient in 12 kidney graft biopsies with TCMR: 7 with and 5 without endothelialitis. TCMR biopsies were selected by an expert nephropathologist to ensure that, while the severity of endothelialitis (v Banff score) differed between the 2 groups of biopsies, the intensity of interstitial (i) and tubular (t) inflammation was similar (i+t: 4.0 ± 0.0 in group without endothelialitis [end^–^] vs. 5.5 ± 0.5 in group with endothelialitis [end^+^], *P* = 0.339; Figure 5D). The CXCL12 gradient was then analyzed using computer-assisted histomorphometry by an independent experimenter blinded to the Banff scores (Figure 5E). In line with the hypothesis, TCMR biopsies without endothelialitis exhibited a preserved chemokine gradient, whereas both the distribution (0.14 ± 0.04 vs. 0.09 ± 0.05; *P* = 0.026) and the intensity (0.99 ± 0.37 vs. 0.70 ± 0.32, *P* = 0.026) of the gradient were dramatically compromised in TCMR biopsies with endothelialitic lesions (Figure 5, F and G). These data suggest that the chemokine gradient is required for the protection of graft endothelium during TCMR.

## Discussion

In the present study, we provide molecular and cellular data confirming that, unlike AMR, in which the graft vasculature is the main target of the alloimmune response, the allogeneic ECs of the graft remained largely preserved from alloreactive cytotoxic T cell–mediated damage during TCMR. Therefore, while both AMR and TCMR are consequences of the response of the recipient’s adaptive immune system to donor-specific alloantigens, the humoral and cellular effector pathways result in diametrically opposed compartmentalization of graft damage (1).

Recent translational studies have demonstrated that the size of immunoglobulins severely restrains their ability to diffuse outside the circulation, explaining why the histological damages of AMR are concentrated in the graft vasculature, which expresses the only accessible alloantigens (2). Conversely, why TCMR lesions spared the ECs of the graft remained unexplained. Indeed, allogeneic ECs of the graft are of donor origin, and alloreactive CTLs establish close interactions with them during their migration from the circulation to the epithelial compartment of the renal allograft. Since the preservation of graft endothelium during TCMR could not be explained by the lack of interaction between effector and target cells, the concept of vascular immune privilege has been proposed (33–35).

Immune privilege is usually seen as the consequence of an intrinsic cellular property allowing the target cells to escape destruction by the immune effectors. A prototypical example of this concept is the eye, which cannot tolerate destructive inflammatory responses. Seminal studies published more than 2 decades ago demonstrated that corneal cells are characterized by the expression of Fas ligand, a type II membrane protein that triggers apoptosis in Fas-bearing cells (including activated lymphocytes), thereby leading to immune tolerance (36). This mechanism has been shown to help control tissue damage in response to viral infection (37) and to protect corneal transplants from rejection (38). In addition to Fas ligand itself (39), various surface molecules have been proposed over the years to alternatively explain the resistance of ECs to T cell–mediated cytotoxicity, including the B7 family molecules programmed death ligands 1 and 2 (PD-L1 and PD-L2) (40) and CD31 (41). Another possible cell-intrinsic mechanism that could explain how ECs escape CD8^+^ T cells may rely on antigen presentation. Indeed, although ECs do expresses MHC molecules (42, 43), they seem to have an impaired capacity to present immunodominant antigenic peptides (33). However, the latter mechanism is unlikely to be involved in TCMR, during which it is the intact allogeneic MHC molecules themselves that are recognized by alloreactive T cells (44).

Our study challenges the traditional view of vascular immune privilege due to intrinsic EC properties. Indeed, endothelial and epithelial cells are equally susceptible to alloreactive cytotoxic T cell–mediated destruction in vitro. The comparison of the dynamics of the interactions of alloreactive CTLs with the 2 types of cellular targets of the graft in vivo revealed that the durations of contact with ECs were shorter than with epithelial cells and resulted in less frequent complete arrests for cytotoxic effectors. Data from intravital microscopy obtained in a murine kidney transplantation model were confirmed by the analysis of kidney graft biopsies with TCMR, which led us to suspect that chemotaxis, i.e., the process of migration of CTLs through the endothelium of the graft, from the circulation to the tubulo-interstitial compartment, might be the explanation for the different fate of endothelial and epithelial targets .

Among the chemokines involved in the recruitment of CTLs is CXCL12, the intracellular signaling of which has been shown to interfere with the TCR pathway (30) and to influence cytotoxic synapse duration (31). Supporting our hypothesis, we found that CTLs migrating along a CXCL12 gradient had a significantly reduced ability to destroy cellular targets, regardless of whether the targets were endothelial or epithelial cells, or whether shear forces from blood flow were present. Notably, in human TCMR biopsies from grafts without endothelialitis, the CXCL12 gradient was preserved, whereas it was significantly disrupted in biopsies with v lesions. This suggests that the key factor was not a direct effect of CXCL12 on CD8^+^ T cell function, but rather the migration process itself. Consistent with this, replacing the CXCL12 gradient with other CXC and CC chemokines produced similar results, whereas exposing CD8^+^ T cells to chemokines without a chemotactic gradient restored their cytotoxicity. This phenomenon may stem from the cytoskeleton’s dual role in both migration (45, 46) and cytotoxic synapse formation (47, 48). Simultaneously confronted with both injunctions, it appeared that the order to migrate overrides to killing for CTLs. Like biathletes, who have to choose whether they will use their muscles to ski or to shoot, it is tempting to speculate that the mobilization of the cytoskeleton to respond to the chemotactic gradient reduces the possibility for alloreactive T cells to establish long and efficient cytotoxic synapses, which in turn protects the endothelium of the graft during TCMR.

Beyond the peculiar situation of TCMR, this original (cell-extrinsic) immune privilege mechanism might be at stake in many other conditions in which the maintenance of vascular integrity is crucial. The need to protect the microcirculation in inflamed sites is indeed critically important in host defense: T cells must purge organs of virus infections without compromising the microcirculation, which would destroy the organ and kill the host. Future work will have to confirm the validity of this hypothesis and universal character of this concept.

## Methods

### Sex as a biological variable 

Patients’ samples from both sexes and were used. Mice of both sexes were used. Sex was not considered as a biological variable in this study.

### Human pathology

#### Histological analyses.

Renal graft biopsies were fixed in acetic acid–formol–absolute alcohol, and paraffin-embedded sections were stained by routine methods.

In some analyses, indirect IHC was performed using anti–human CD8 (Dako, catalog GA623) and CD34 (MilliporeSigma, catalog HPA036723) or COL-IV (MilliporeSigma, catalog SAB4500369) antibodies. TCMR pathology samples were cropped, and regions with no cellular infiltrate were discarded. The different images were then computed in a semiautomated python pipeline to quantify CD8^+^ T cell density within the vascular and tubular compartments, using the scikit-image python module. Briefly, a color deconvolution algorithm was applied to separate the RGB images into 3 (hematoxylin, eosin, and DAB [detection antibody]) channels. The hematoxylin channel was then processed by applying thresholding (Otsu method), followed by a sequence of binary erosion/dilation steps to obtain either vascular (CD34 staining) or tubular (COL-IV staining) regions of interest (ROIs). A manual correction of the ROIs was performed. CD8^+^ T cells were automatically counted using a Laplacian of Gaussian blob detection and were then filtered, keeping only cells within the ROI. The average CD8^+^ T cell density per TCMR sample was defined as the ratio of the number of detected CD8^+^ T cells to the area of each ROI. Since glomeruli, whose capsules express COL-IV, could be wrongly recognized as tubular regions, a manual correction of the ROIs was performed.

#### EndMT quantification in human graft biopsies.

EndMT marker quantification was performed as previously described (14). IHC was used to detect EndMT markers in paraffinized human tissue. Target retrieval was carried out by heating the tissue in citrate buffer. The sections were incubated overnight at 4°C with PBS containing mouse anti–fascin 1 (Dako), anti-vimentin (Diagnostic Biosystems), or anti-hsp47 (Stressgen) antibodies. The immunoreactive proteins were then visualized with anti-mouse Histofine Simple Stain MAX PO and AEC + substrate – chromogen (Dako). As a negative control, the primary antibody was replaced by an equal concentration of mouse IgG. The quality of the staining for each experiment was controlled by comparison with a positive (ABMR) and a negative (normal kidney) case. Double staining of 3 EndMT markers with the endothelial marker CD34 was done according to the polink DS-MM kit (GBI Labs). Each EndMT marker was assessed 3 separate times in the EC of the peritubular capillaries (PTCs) (identified using a CD34-based mask) by a researcher blinded to the clinical data. The semiquantitative assessment was scored as follows: no staining: 0; strong PTC cell staining in less than 10% of cells: 1; in 10%–24%: 2; 25%–50%: 3; greater than 50%: 4. Unless specifically mentioned for an individual marker, the highest of the 3 markers defined the EndMT score. Grafts with a score of 2 or higher (10% or more of PTC cells were strongly stained by any 1 of the 3 markers) were considered EndMT^+^. This cutoff was defined before any statistical analysis, based on our experience with EMT markers and the level of EndMT marker expression observed in normal kidneys.

#### Pathologic analyses of the chemokine gradient in human biopsy specimens.

TCMR biopsy specimens were selected from the biocollection of Necker Hospital (Paris, France) by an expert nephropathologist. The samples were categorized into 2 groups on the basis of the presence of endothelialitic (v score) lesions. To ensure comparability between groups, cases were selected with matched severity of interstitial inflammation (i score) and tubulitis (t score) according to Banff criteria. All specimens underwent standard histological processing, including H&E staining and immunohistochemical staining for CXCL12 using DAB.

Whole-slide images (WSIs) were digitized using a high-resolution scanner and processed through a custom automated image analysis pipeline (Python) to quantify the CXCL12 staining patterns. The WSIs were first divided into tiles of 2,048 × 2,048 pixels, filtering out tiles with excessively large empty areas (>50% of tile area). Each tile underwent HED (H&E-DAB) color deconvolution, and tissue segmentation was achieved by adaptive thresholding on the hematoxylin channel. CXCL12^+^ regions were identified through thresholding of the DAB channel using a threshold value of 0.035. CXCL12^+^ regions were analyzed, with quantification of their area, mean staining intensity, and spatial distribution. Measurements from all tiles were aggregated to obtain whole-slide metrics. CXCL12^+^ surface area and staining intensity were normalized to total tissue area.

### Transcriptomics analyses of human graft biopsy specimens 

Transcriptomics data were downloaded from the Gene Expression Omnibus (GEO) website (https://www.ncbi.nlm.nih.gov/geo/) using the accession numbers GSE36059 and GSE48581 (rejection microarray data); GSE109564 (rejected kidney allograft scRNA-Seq data); and GSE118184 (healthy donor kidney scRNA-Seq data).

All transcriptomics analyses were performed using R, version 3.6.2. scRNA-Seq data from a healthy donor kidney were processed using the Seurat package, version 5, as described previously (11). Briefly, the publicly available unique molecular identifier (UMI) count matrix was filtered, retaining only genes found to be expressed in more than 10 cells. The differential gene expression matrix was then scaled by total UMI counts and log-normalized with a 10,000-scale factor. Cells with more than 0.3% of mitochondrial genes or fewer than 300 or more than 4,000 detected genes were discarded, resulting in 4,392 cells. Highly variables genes were identified using the “MeanVarPlot” selection method of the “FindVariableFeatures” function. *t*-Distribution stochastic neighbor embedding (*t*-SNE) was performed and the “FindCluster” function was used with a resolution parameter of 0.6, which permitted the identification of 16 cell clusters. These cell clusters were annotated as ECs, tubular epithelial cells (distal convoluted tubule [DCT] cells, intercalated cells [ICs], loop of Henle [LH] cells, principal cells [PCs], PT cells), or other cell types (podocytes, immune or undefined cells) using literature-reported marker genes. Endothelial (*n* = 84) and tubular epithelial (*n* = 800) lineage genes were extracted from each annotated cluster gene list and used for downstream GSEA. The annotated lineage gene list is provided in the Supplemental Data File 1.

Single-cell transcriptomics data from rejected kidney transplants were also from the BioStudies database (accession E-MTAB-12051), with data from 3 AMR, 4 NR, and 1 TCMR biopsies (49, 50). Raw count matrices were filtered to include cells with at least 200 features and genes present in at least 3 cells. Data were normalized using the LogNormalize method with a scale factor of 10,000. Variable features were identified using the variance-stabilizing transformation (VST) method, selecting the top 2,000 most-variable genes. After scaling the data, principal component analysis (PCA) was performed on the variable features. On the basis of an elbow plot approach, the first 10 principal components were used for downstream analysis. Graph-based clustering was performed using the “FindNeighbors” and “FindClusters” functions with a resolution parameter of 0.1, which identified 8 clusters. UMAP was used for dimensionality reduction and visualization. ECs were identified on the basis of PECAM1 marker gene expression and extracted for further analysis. Differential gene expression analysis between conditions (NR vs. AMR and NR vs. TCMR) was performed using the Wilcoxon rank-sum test. Genes with adjusted *P* values of less than 0.05 and absolute log_2_ fold changes of greater than 2.5 were considered significantly differentially expressed. GSEA was conducted using the fgsea package against the Reactome pathway database. Genes were ranked according to their log_2_ fold changes, and pathways related to cell damage mechanisms (including the terms “apoptosis,” “necrosis,” “cell stress,” “DNA damage,” “inflammation,” “mitochondrial dysfunction,” “autophagy,” and “ferroptosis”) were specifically analyzed. Normalized enrichment scores (NESs) were calculated to determine pathway activation in AMR and TCMR conditions compared with NR controls.

Microarray data were processed as previously described (12). Briefly, CEL files (corresponding to 105 AMR, 67 TCMR, and 501 NR kidney biopsies) were imported using the oligo package. Twelve mixed-rejection biopsies were excluded from the dataset. Robust multichip average (RMA) normalization and linear regression were performed using the limma package. Transcriptomics profiles of “pure” AMR and TCMR samples were established using NR biopsies as a reference. The most differentially expressed genes (DEGs) in AMR and TCMR samples by comparison with NR kidneys were identified using the Empirical Bayes statistic method (absolute log fold change >0.2 and adjusted *P* <10^–8^). The DEG lists were then filtered out using the endothelial and tubular epithelial lineage genes, giving a list of DELGs associated with AMR or TCMR.

### In silico modeling of TCMR histology 

An in silico model of TCMR histological analysis was developed in python (3.9.0) language using the “tkinter” and “shapely” modules. Briefly, 2 compartments — red and blue — of predefined areas were randomly drawn on an empty canvas. Variable numbers of circular cells animated with random speed and direction were then added to the canvas. When a cell encounters 1 of the 2 compartments, it freezes for a given contact duration before restarting in a random direction and at a random speed. The number of cells immobilized in each region over pseudotime was recorded under different conditions (cell numbers, compartment relative size areas, relative contact durations of cells with both compartments). Each run was recorded in triplicate using different random seeds to ensure comparability between variable conditions. Cell density was determined as cells per square millimeter by calculating the ratio of immobilized cells per compartment to the area of each compartment, scaled on a cell diameter of 8 μm (average CD8^+^ T cell size).

### In vitro experiments

#### Human cell preparation and cultures.

The human conditionally immortalized glomerular endothelial EC line (ciGENC) (19) was provided by S.C. Satchell (University of Bristol, Bristol, United Kingdom). The cells were cultured in EC growth medium 2 (PromoCell, catalog C-22211) at 33°C in 5% CO_2_ for maintenance and were put at 37°C in 5% CO_2_ for 7 days to allow differentiation before being used for cytotoxicity experiments. β2-Microglobulin–KO ciGENC cells were provided by Dany Anglicheau and Baptiste Lamarthée (Necker Hospital, Paris, France).

The human PT epithelial cell line HK-2 was provided by N. Pallet (Université Paris Descartes, Paris, France) and was cultured in DMEM enriched with ITS Liquid Media Supplement (MilliporeSigma, catalog 3146), human EGF (200 ng/mL, MilliporeSigma, catalog E9644), hydrocortisone (500 ng/mL, MilliporeSigma, catalog H0888-1G), tri-iodothyronin (4 pg/mL, MilliporeSigma, catalog T5516), penicillin/streptomycin (Gibco, Thermo Fisher Scientific), and 1% FCS.

The SAL-A2 cell line, corresponding to the HLA-A2–transfected K-562 cell line, was provided by I. Doxiadis and S. Heidt (University of Leiden, Leiden, Netherlands).The cell line was cultured in RPMI-1640 (Thermo Fisher Scientific) complemented with FBS 10% (Dutscher), l-glutamine 2 mM (Thermo Fisher Scientific), penicillin/streptomycin, and 25 mM HEPES (Thermo Fisher Scientific) (referred to hereafter as complete RMPI medium), combined with geneticin 1.75 mg/mL (G418 sulfate, Thermo Fisher Scientific).

#### Generation of human cytotoxic effector cells.

CD8^+^ T cells were purified by negative selection (Magnetic Enrichment Kit, STEMCELL Technologies, catalog 17953) from PBMCs obtained from an HLA-A2–negative healthy donor, stained with CellTrace Violet (CTV) (Thermo Fisher Scientific, catalog C34571), and cocultured with 100 Gy irradiated, CFSE-stained SAL-A2 cells (1:1 ratio) in X-VIVO 20 medium (Lonza) complemented with 10% human AB serum and recombinant human IL-2 (100 IU/mL, R&D Systems). Unspecific CD8^+^ effector T cells were generated by replacing SAL-A2 cells with CD3/CD28 dynabeads (1 bead/1 cell ratio, Thermo Fisher Scientific, catalog 11161D) that were magnetically removed after 48 hours. After 6 days of coculturing, the cells were incubated with a fixable viability dye (Thermo Fisher Scientific) and stained using anti-CD8 (clone SK1, 1/10, BioLegend) and anti-CD4 (clone SK3, 1/10, BD Biosciences) antibodies, and then proliferating CD8^+^ T cells were sorted by flow cytometry, gated as live CFSE^neg^CD4^neg^CD8^pos^ and CTV^neg^ cells. These cells were thereafter used in cytotoxicity in vitro experiments.

#### In vitro cytotoxicity assays with human cells.

Impedancemetry was performed, in which differentiated ciGENC or HK2 cells were seeded on a gelatin-coated E-Plate VIEW 96 PET (Agilent Technologies), allowing impedance measurement, and then incubated at 37°C in 5% CO_2_ (10^4^ cells/well). After 24 hours, either allospecific or unspecific CD8^+^ effector T cells at various effector/target ratios were added to the culture, with recombinant human IL-2 (100 IU/mL, R&D Systems).

Adherent target cell viability was monitored every 5 minutes for 10 hours by electrical impedance measurement with an xCELLigence RTCA SP instrument (ACEA Biosciences). The cell indices were normalized to the reference value (measured just prior to adding effector cells to the culture). EC viability in the experimental well was normalized over the control wells with unspecific CD8^+^ effector T cells.

For time-lapse microscopy, differentiated ciGENC cells were stained with CTV, seeded on a gelatin-coated, 384-well microplate (PerkinElmer), and then incubated at 37°C in 5% CO_2_ (5 × 10^3^ cells/well). After 24 hours, either allospecific or unspecific CD8^+^ effector T cells were stained using CM-DiI Dye (Thermo Fisher Scientific) and added to the culture in phenol red–free complete RPMI with recombinant human IL-2 (100 IU/mL, R&D Systems) and annexin V Alexa Fluor 647 Ready Flow Conjugate (Thermo Fisher Scientific).

Imaging was performed on a Zeiss AxiObserver Z1 inverted fluorescence microscope, acquiring 1 image every 40 minutes for 24 hours. The cells were excited at 430 nm (CTV-stained target cells), 560 nm (CM-DiI–stained effector cells), and 650 nm (Alexa Fluor 647–annexin V ), using the bright-field channel.

Cell viability was assessed visually according to the appearance of annexin V staining in target cells and assessed in an automated manner by evaluating the level of confluence of CTV-stained target cells.

Transwell assays were performed, in which ciGENC or HK-2 cells were seeded on gelatin-coated, 5 μm pore, 6.5 mm cell culture inserts and incubated at 37°C in 5% CO_2_. After 24 hours, the cells were stained with calcein AM (Thermo Fisher Scientific, catalog L3224), and confluence was assessed before adding the effector T cells by imaging the cell layer on an epifluorescence microscope at 488 nm. Complete RPMI medium (600 μL), complemented with or without 0 ng/mL, 80 ng/mL, or 320 ng/mL CCL2, CXCL9, or CXCL-12 chemokines (PeproTech), was added to the lower chamber of the Transwell plate. Either allospecific or unspecific CD8^+^ effector T cells were then added to the upper chamber and cocultured with target cells at 37°C in 5% CO_2_. After 6 hours, cell culture inserts were removed from the plate, and cell viability was determined through automated confluence assessment using epifluorescence microscopy. Confluence measurements were performed using ImageJ (NIH) and normalized over a condition with no effector T cell.

#### In vitro hemodynamics flow model.

ciGENCs were seeded into microfluidic channels (μ-slide VI0.4, Ibidi) precoated with extracellular matrix (100 μg/mL type I collagen from rat tail, Gibco, Thermo Fisher Scientific), with or without supplementation of CXCL12 (R&D Systems, 350-NS, 80 ng/mL). ciGENCs were cultured at 33°C until confluence, and then at 37°C for 5 days to induce differentiation, using complete EC growth medium (Lonza, EGM-2 MV, CC-3202).

Once differentiated, ciGENCs were subjected to gradually increasing laminar shear stress (ranging from 1–5 dyne/cm²) for 48 hours. To simulate the inflammatory conditions associated with rejection, cells were then exposed to recombinant human IL-1β (R&D Systems, 201-LB-010, 10 ng/mL) for the final 15 hours.

Human activated CD8^+^ T cells (either allospecific or non-allospecific) were thawed and preincubated for 15 hours in RPMI medium supplemented with 10% FBS and 200 U/mL recombinant human IL-2 (R&D Systems, 202-IL-050). These CD8^+^ T cells were subsequently introduced into the microfluidic system at a shear stress of 0.1 dyne/cm². Their interactions with activated ciGENCs, along with morphological changes in ECs, were monitored in real time via video microscopy (AxioObserver, Zeiss) over a 10-minute period. Image analysis was performed using Fiji software (ImageJ, NIH).

### In vivo experiments

#### Mouse model of T cell–mediated kidney graft rejection and surgical procedures.

B6 (C57BL/6J; Thy1.2, CD45.2, H-2B) mice; B6.PL-Thy1a/CyJ mice (Thy1.1, CD45.2, H-2B) mice; B6-OVA (C57BL/6J-Tg[CAG-OVA]916Jen/J; CD45.2, H-2B) mice; and OT-I (C57BL/6-Tg[TcraTcrb]1100Mjb/J; CD45.2, H-2B) mice were purchased from The Jackson Laboratory. P14 mice (B6.Cg-Tcratm1Mom-Tg[TcrLCMV]327Sdz; CD45.2, H-2B) were purchased from Taconic. Transplantation of vascularized kidney grafts was performed as previously described (51).

#### In vitro generation of CTLs for in vivo mouse experiments.

OVA-specific CTLs were generated. Briefly, DCs were generated by culturing bone marrow cells with IL-4 and GM-CSF (Peprotech) for 8 days. Next, DCs were stimulated with 100 ng/mL LPS overnight and pulsed with either 10 μg/mL OVA (SIINFEKL) or 5 μg/mL LCMV (KAVYNFATM) peptides (GenScript) for 2 hours at 37°C. OVA or LCMV peptide–pulsed DCs (2 × 10^6^ to 3 × 10^6^) were injected i.v. with 5 × 10^5^ OT-I (Thy1.1, CD45.2) or P14 (Thy1.2, CD45.2) T cells, respectively, into B6 (Thy1.2, CD45.1) mice, according to previously published methods (52). Five days later, splenic and lymph node cells were labeled with 2 μM CFSE or CellTracker Orange (CTO), and OT-I and P14 effector cells were recovered via high-speed sorting by gating on CD45.2^+^CFSE^+^CTO^+^ cells using the following dump channel: CD4^+^CD45R/B220^+^CD11c^+^CD11b^+^CD49b^+^Ly^–^76^+^CD16/32^+^F4/80^+^. Flow analysis confirmed that sorted cell populations were more than 95% CD8^+^CD44^+^ and were OVA- or LCMV–MHC-I tetramer^+^. OT-I and/or P14 effector cells were then cotransferred i.v. (7 × 10^6^ to 10 × 10^6^ each) into B6 (Thy1.2, CD45.1) recipients of B6-OVA kidney grafts.

#### Bi-photon intravital microscopy and image analysis.

Bi-photon intravital microscopy was performed on transplanted kidneys using a previously established method (24). Image analysis was performed by Python scripting. Briefly, cells were detected and 3D located at each time point, and kinetic parameters were determined in an automated manner using the Trackpy Python module. The intra- or extravascular position was determined manually for each cell detected by the analysis program. The arrest coefficient was calculated as the percentage per track that a cell moved slower than 2 μm/min.

### Statistics

Statistical analyses were conducted using R (version 4.4.1). Continuous variables are presented as the median ± IQR if not otherwise specified. A 2-sided Student’s *t* test was used to compare normally distributed continuous variables, and a nonparametric Mann-Whitney *U* test was applied for non-normally distributed variables, with a 0.05 significant threshold. Bonferroni’s correction was used for multiple comparisons. Unless otherwise specified, data are presented as mean ± SEM.

### Data availability

The values corresponding to all data points shown in graphs and values behind any reported means are available in the Supporting Data Values Excel file.

### Study approval

TCMR biopsy specimens were retrospectively selected from the biocollections of Tenon and Necker hospitals (both from Assistance Publique Hopitaux de Paris) and the Hospices Civils de Lyon, in compliance with French regulations on biomedical research. All animal studies were approved by the University of Pittsburgh IACUC (protocol no. 12050385; PHS assurance no. A3187-01).

### Data and materials availability

All data are available in the main text of the supplemental materials. Code is available upon request to the corresponding author.

## Author contributions

TB and OT conceptualized the study. TB, MO, GF, CG, MR, CR, LM, VM, CS, VD, NP, DA, BL, A Hertig, AN, JPDVH, and YXD performed experiments. TB and OT wrote the manuscript. ST, CR, AK, EM, A Hot, HP, TD, AN, JPDVH, and FGL reviewed the manuscript.

## Supplementary Material

Supplemental data

Supplemental data set 1

Supplemental video 1

Supplemental video 2

Supplemental video 3

Supplemental video 4

Supporting data values

## Version 1 05/28/2025

In-Press Preview

## Version 2 07/15/2025

Electronic publication
