# Supplementary material for: Chemotaxis overrides the killing response in alloreactive CTLs, providing vascular immune privilege during cellular rejection
Source: J Clin Invest. 2025 May 22;135(14):e155191. doi: 10.1172/JCI155191 (PMC12259268; doi:10.1172/JCI155191)
Supplement: Supplemental data [file jci-135-155191-s208.pdf]

SUPPLEMENTAL FIGURES

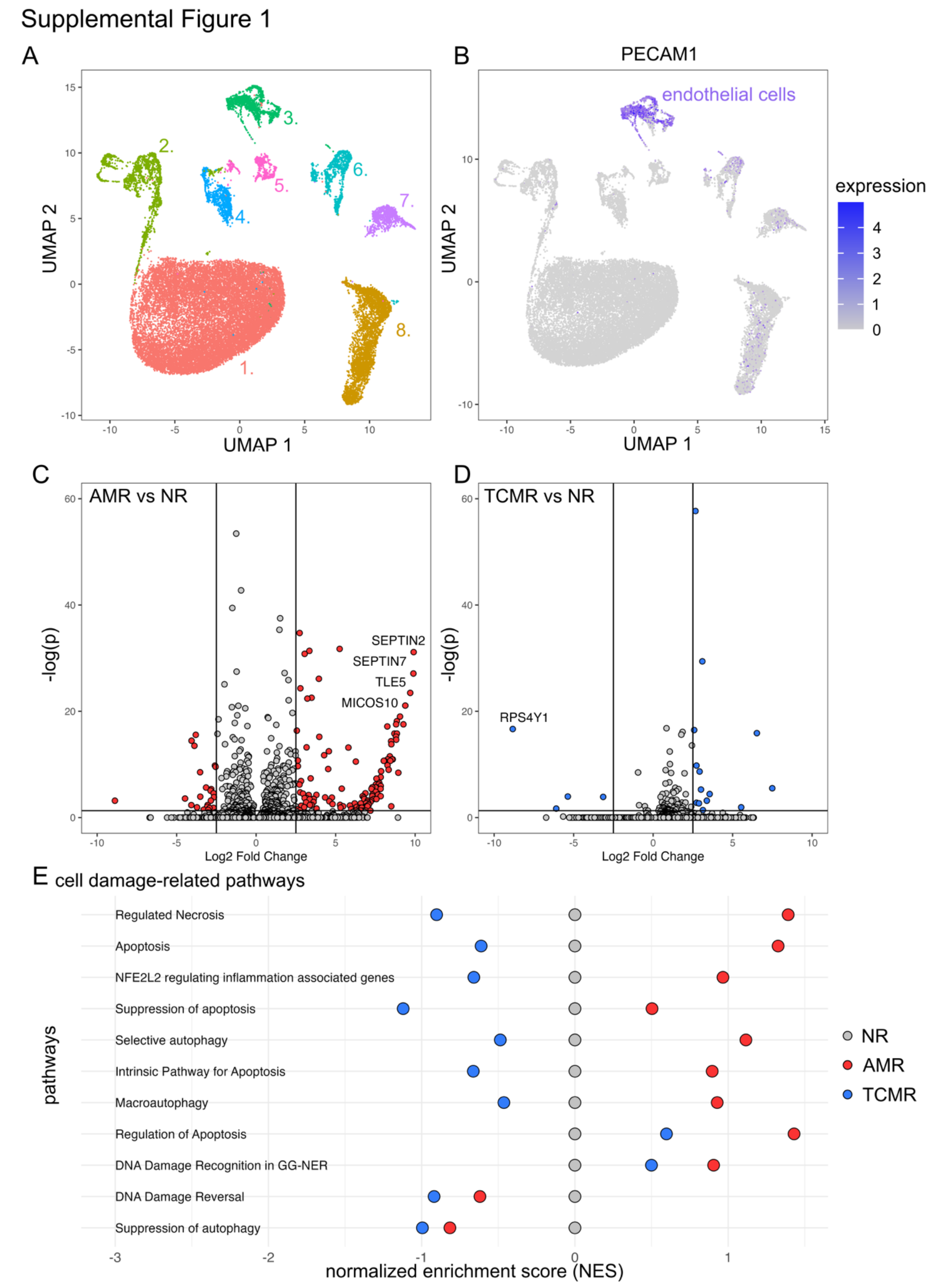

Supplemental Figure 1

Single-cell RNA sequencing analysis of 27,412 cells derived from 8 kidney graft biopsies: 3 antibody-mediated rejection (AMR), 4 normal rejection-free (NR), and 1 T cell-mediated rejection (TCMR). **A.**

UMAP analysis was performed to reduce dimensionality, revealing eight distinct clusters. **B.** UMAP visualization overlaid with the relative expression of the endothelial marker PECAM-1, with color intensity corresponding to average scaled gene expression.
C - D. Volcano plots illustrating differential gene expression in graft endothelial cells during AMR (C) and TCMR (D) compared to NR.
E. Gene Set Enrichment Analysis (GSEA) focusing on cell damage-related pathways in graft endothelial cells for AMR (red) and TCMR (blue), with NR (grey) used as the reference group.

Supplemental Figure 2

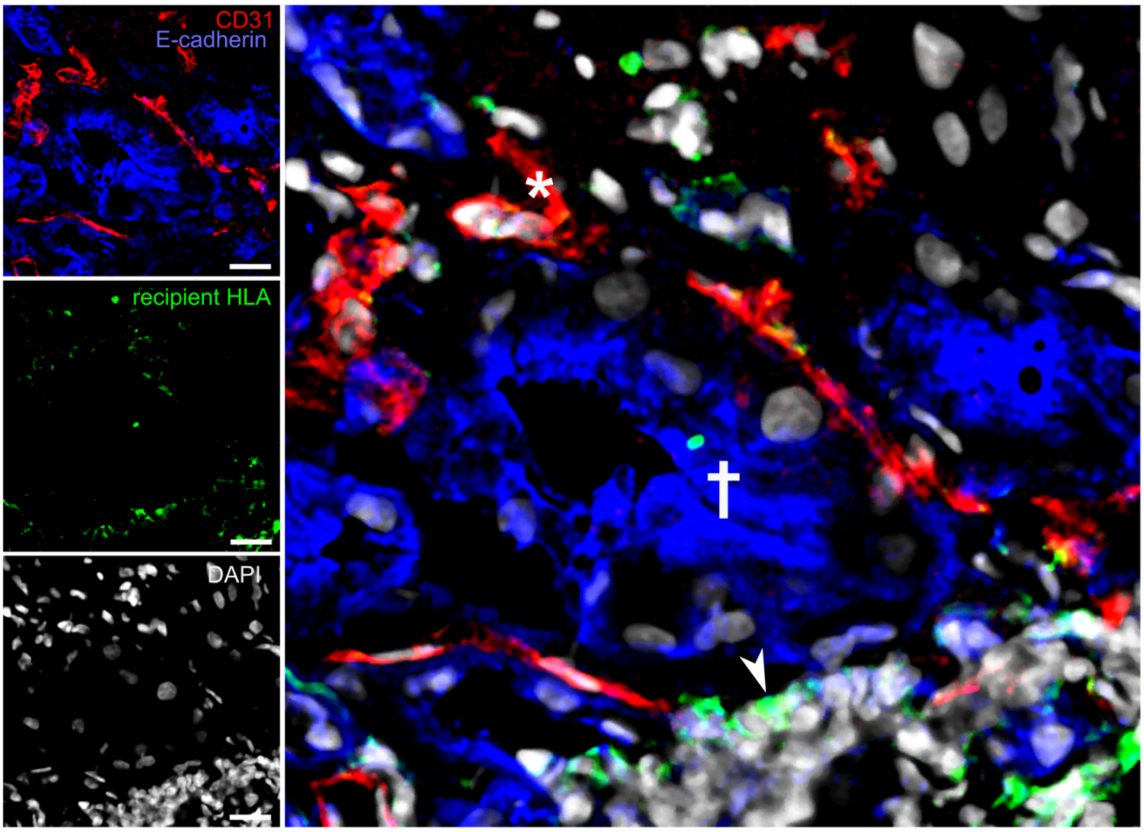

Supplemental Figure 2
Confocal microscopy analysis of a renal allograft biopsy with TCMR. The expression of the recipient-specific mismatched HLA-A24 molecules (green) by graft microvasculature (CD31, red; star), graft tubules (E-cadherin, blue; cross), and infiltrating immune cells from recipient (positive control, arrowhead).

Supplemental Figure 3

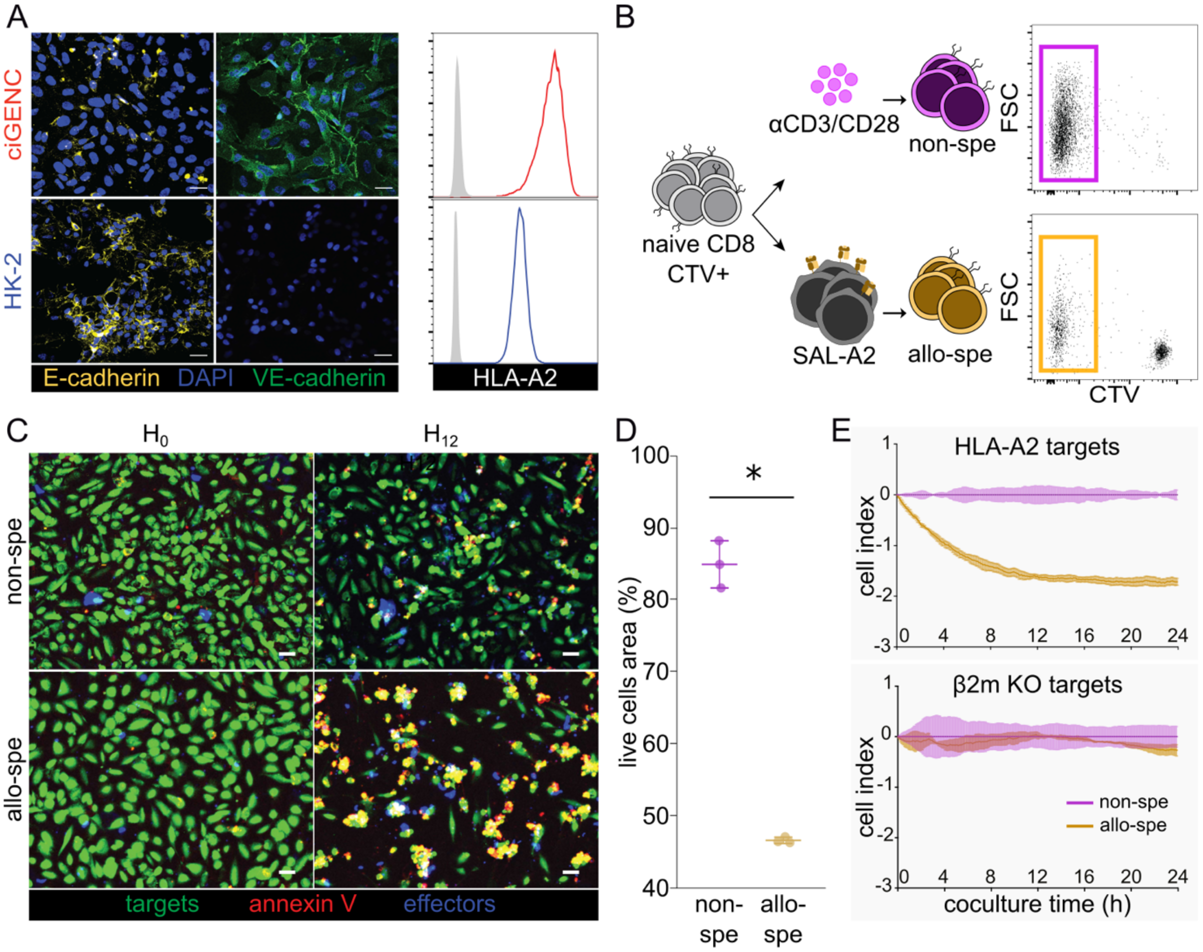

Supplemental Figure 3

A. Confocal microscopy analyses of human cell lines. The glomerular endothelial cell line (ciGENC) expressed the endothelial marker VE-cadherin (green), while the proximal tubule epithelial cell line (HK-2) expressed the epithelial marker E-cadherin (yellow; scalebar = 30µm). Both CiGENC and HK-2 cells expressed the HLA-A2 molecule (right histograms).

B. Schematic representation of the HLA-A2 specific CTLs generation procedure. Naive human CD8+ T cells were stained with CTV and cocultured for 6 days with either 100 Gy-irradiated CFSE stained SAL-A2 cells or anti-CD3/CD28 beads. At the end of coculture, proliferating CD8+ T cells were sorted by cytometry based on the CTV fluorescence.

C – D. Assessment of cytotoxicity and specificity of in vitro generated non-specific (purple) and allospecific (orange) CTLs. (C) Destruction of CTV-stained adherent endothelial cellular targets (green) by non-specific (upper row) or allospecific (lower row) CM-DiI-stained CTLs (blue) was monitored by the decrease of green area and confirmed by the acquisition of the apoptosis marker Annexin V (red) using time-lapse microscopy. Representative images from the begining (left column) and the end (right column) of cocultures are shown (scalebar = 50 µm). (D) Quantification of cell destruction from two independent time-lapse experiments; unpaired two-sample Wilcoxon test.

E. Destruction of HLA-A2+ adherent endothelial cellular targets (upper panel) and HLA-I deficient (β2-microglobulin KO, lower panel) by non-specific (purple, control) and allospecific (orange) CTLs was measured using real-time impedance monitoring. Decrease impedance in the culture well indicated death of adherent cellular targets. Impedance values were normalized over control conditions.

Supplemental Figure 4

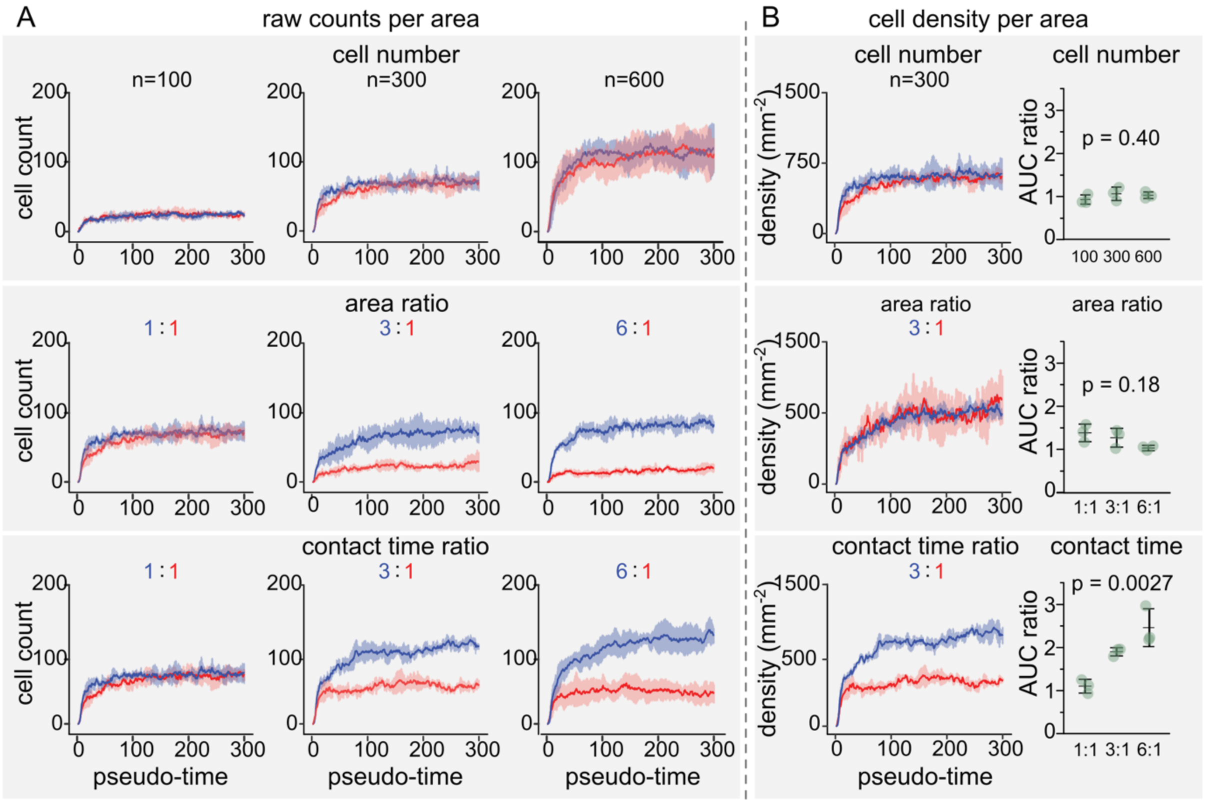

Supplemental Figure 4

In silico modeling of CTLs repartition among the vascular and tubular compartments of the graft. **A.** Raw counts and **B.** density (raw count normalized on area) of CTLs trafficking in randomly generated compartments were plotted over pseudo-time, whilst varying different parameters: number of trafficking cells (upper row), relative sizes of the 2 compartments (middle row), and the relative contact times between the cells and each compartment (lower row). One-way ANOVA.

Supplemental Figure 5

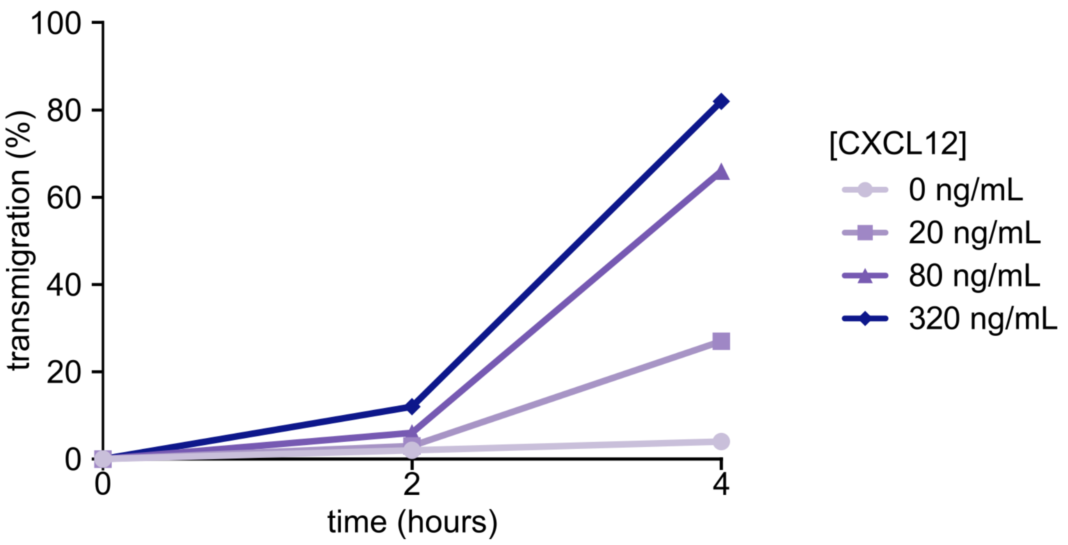

Supplemental Figure 5
Kinetic of CTLs transmigration across a monolayer of CiGENC cells seeded on a 5µm-pored cell culture insert with increasing CXCL-12 gradients.

Supplemental Figure 6

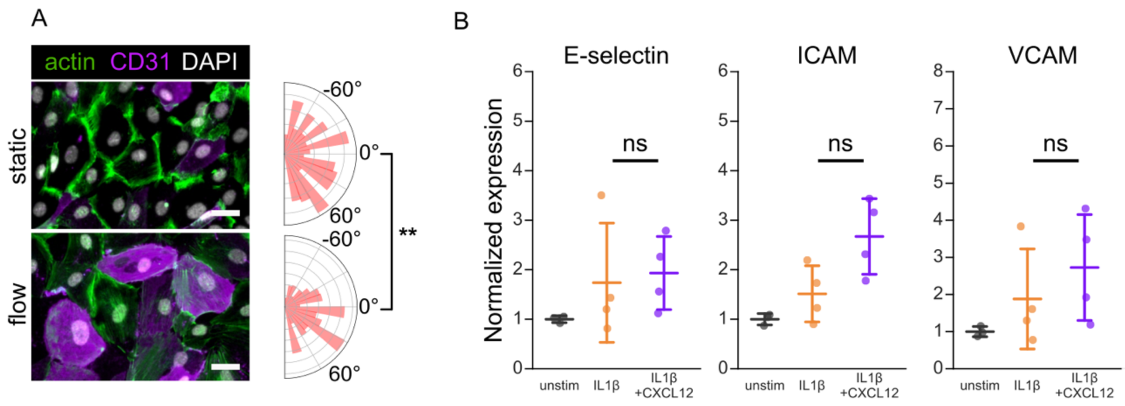

Supplemental Figure 6
CiGENC cells were cultured to confluence in microfluidic channels on an extracellular matrix, with or without CXCL12. Cells were exposed to flow-induced tangential shear stress (flux, lower row) or maintained under static conditions (upper row) for 48 hours.
A. At the end of the culture period, cells were stained for actin (green), CD31 (purple), and nuclei were counterstained with DAPI (white). Cell alignment under flow conditions was compared to static conditions (chi-square test) ; Representative images are shown (scale bars = 20 µm).
B. At the end of the culture, cells were stimulated or not (unstim) with IL-1β and the level of expression of E-selectin, ICAM-1, and VCAM-1 was compared in the various culture conditions.

SUPPLEMENTAL MOVIES

Supplemental Movies #1 to 3

Representative intravital microscopy movies of OVA-specific (OT-I, yellow) and control P14 (cyan) cells trafficking within B6-OVA renal allograft. The vascular compartment is identified by a fluorescent dextran (red).

Movie #1: Intra-vascular OT-I cell
Movie #2: Intra-vascular P14 cell
Movie #3: Extra-vascular OT-I and P14 cells

Supplemental Movie #4

In silico model of CTLs trafficking among randomly generated compartments, representing vascular (red) and tubular (blue) compartments of an allograft respectively. The impact of total cell count (upper row), relative sizes of the 2 compartments (middle row) and relative contact times of trafficking cells with the compartments (lower row) on cell repartition is evaluated over pseudo-time and shown in Supplemental Figure 4.

SUPPLEMENTAL FILES

Supplemental File #1

Annotated gene list obtained from clustering analysis of a healthy donor kidney single-cell RNA seq data (GSE118184). Clustering analysis identified 16 cell clusters that were annotated as “endothelial” or “tubular epithelial”. Genes specifically expressed by each cell types (i.e. “lineage genes”) were used for the downstream analyses of microarray data from graft biopsies with different types of rejection.

Abbreviations are: PT: proximal tubule, DCT: distal convoluted tubule, IC: intercalated cells, LH: loop of Henle, PC: principal cells.
